# Concomitant anti–tumor necrosis factor and anti–interleukin 17 therapy in severe, refractory hidradenitis suppurativa: A case series

**DOI:** 10.1016/j.jdin.2026.08.004

**Published:** 2026-08-28

**Authors:** Estella Bastian, Lia Bizuneh, Erin H. Amerson, Haley B. Naik

**Affiliations:** a Department of Dermatology, University of California, San Francisco, San Francisco, California; b Case Western Reserve University School of Medicine, Cleveland, Ohio; c Zuckerberg San Francisco General Hospital, San Francisco, California

**Keywords:** adalimumab, biologics, dual therapy, efficacy, epidemiology, hidradenitis suppurativa, infliximab, safety, secukinumab

*To the Editor:* Anticytokine biologics, including anti–tumor necrosis factor (TNF) and anti–interleukin 17, are approved for moderate-to-severe hidradenitis suppurativa (HS) despite demonstrating limited clinical response rates of 42% to 59%.^1^, ^2^, ^3^ Dual biologic therapy targeting multiple inflammatory pathways may improve disease control in treatment-refractory HS.^4^^,^^5^

We retrospectively evaluated the therapeutic responses and safety of concomitant anti-TNF and anti-interleukin 17 therapy in 11 patients with physician-confirmed treatment-refractory HS, who received at least 3 months of continuous dual therapy between 2022 and 2025. Treatment-refractory HS was defined as the development of new HS lesions in new locations despite the maximum available dose of biologic monotherapy for at least 4 months. Demographics, medical history, patient- and physician-reported outcomes, and adverse events were obtained from chart review. Baseline was defined as a dermatologic visit within 6 months before dual biologic initiation; the end point was the most recent visit. The primary outcome was HS-Physician Global Assessment reduction. We report descriptive statistics and comparisons using the Wilcoxon signed-rank test. This study was approved by the University of California, San Francisco Institutional Review Board.

Eleven patients (8 women and 3 men) were included, with a median age of 34.0 years (IQR, 24.5-45.5) and a median body mass index of 31.0 kg/m^2^ (24.4-38.5) (Table I). All had severe, longstanding disease. Patients had tried 3 prior biologic monotherapy trials on average, including repeated trials of the same agent; 5 had received ≥3 different agents.

**Table I:** Baseline characteristics of patients receiving dual biologic therapy

Total patients, *n*	11
Women, *n* (%)	8 (72.7%)
Race/ethnicity, *n* (%)	
Black	7 (63.6%)
White	2 (18.2%)
Mixed race∗	1 (9.1%)
Hispanic or Latine	1 (9.1%)
Age, y (median, IQR)†	34.0 (24.5-45.5)
BMI, kg/m^2^ (median, IQR)†	31.0 (24.4-38.5)
HS history	
Age at onset, y (median, IQR)	19 (13.75-29)
Disease duration, y (median, IQR)	11 (5-16)
Baseline disease severity	
Hurley stage, *n* (%)	
Hurley stage 2	1
Hurley stage 3	10
HS-PGA (median, IQR)†	4 (4-4.8)
No. active body sites (median, IQR)†	5 (4-6)
Treatment history, *n* (%)	
Systemic antibiotics	11 (100%)
IV Ertapenem	8 (72.7%)
Systemic corticosteroids	7 (63.6%)
Hormonal agents (women, *n =* 8)	6 (75.0%)
Isotretinoin	4 (36.4%)
Metformin	3 (27.3%)
Surgery‡	8 (72.7%)
Biologic monotherapy, *n* (%)	
No. of previous biologic monotherapy trials (median, IQR)	3 (2-4)
Adalimumab	9 (81.8%)
Infliximab	11 (100%)
Secukinumab	3 (27.3%)
Other§	2 (18.2%)
Comorbidities, *n* (%)	
Mood disorders	9 (81.8%)
Diabetes	4 (36.4%)
Dyslipidemia	4 (36.4%)
History of tobacco smokingװ	4 (36.4%)
Hypertension	3 (27.3%)
Pilonidal cyst	5 (45.5%)
Nodulocystic acne	3 (27.3%)
Dissecting cellulitis	2 (18.2%)
Psoriasis¶	3 (27.3%)

*BMI*, Body mass index; *HS-PGA*, Hidradenitis Suppurativa Physician Global Assessment; *IV*, intravenous.

∗ Patient identified as American Indian/Alaska Native, Asian, and Black/African American.

† Determined at the time of dual therapy initiation.

‡ HS deroofing and excisions.

§ One patient trialed etanercept and ustekinumab monotherapies, as well as dual infliximab and tofacitinib. Another patient trialed tofacitinib and ixekizumab monotherapies.

װ None were active smokers.

¶ Two of 3 were diagnosed with tumor necrosis factor–induced psoriasis on biologic monotherapy.

Median dual biologic treatment duration, determined at the end point visit, was 6.2 months (range, 3.1-28.6 months); 2 patients received dual therapy for >1 and 2 years, respectively (Table II). Ten patients remained on dual therapy at the end point. Median HS-Physician Global Assessment decreased by 2 points (1-3.5, *P* = .002); 6 patients achieved ≥2-point reduction (Supplementary Figure 1, available via Mendeley at https://data.mendeley.com/datasets/r69bks7brn/1). Median number of active body sites decreased by 3 (1-4, *P =* .008). Most patients had reductions in inflammatory lesions (9/10), drainage (8/10), and pain and/or subjective disease activity (8/10). Median pain score reduction was 3 points (1.25, *n =* 4). One patient discontinued therapy due to xerosis and eczematous eruption after bimekizumab initiation.

**Table II:** Concomitant biologic regimens, clinical response, and safety outcomes

Concomitant biologic regimens,∗ n = 11 (%)	
Infliximab (10-15 mg/kg q4w) + secukinumab (300 mg q4w-q1w)	8 (72.7%)
Infliximab (10-12.5 mg/kg q4w) + bimekizumab (320 mg q4w-q2w)	2 (18.2%)
Adalimumab (80 mg q1w) + secukinumab (300 mg q2w)	1 (9.1%)
Duration of dual biologic therapy, mo, *n =* 10 (median, IQR)	6.2 (4.6-7.7)
Physician-reported treatment response, *n =* 10 (%)	
Median reduction in HS-PGA (IQR, *P*)	2 (1-3.5, 0.002)
Two-point reduction in HS-PGA	6 (60%)
Median reduction in the no. of active body sites (IQR, *P*)	3 (1-4, 0.008)
Reduction in inflammatory lesions	9 (90%)
Reduction in drainage	8 (80%)
Patient-reported treatment response, *n =* 10 (%)	
Patient-reported reduction in pain and/or disease activity	8 (80%)
Adverse events, *n =* 11 (%)	
Rash†	1 (9.1%)
Upper respiratory infection	2 (18.2%)
Aphthous ulcer	1 (9.1%)
Oral thrush‡	1 (9.1%)
Psoriasis, psoriasiform dermatitis	2 (18.2%)

*HS-PGA*, Hidradenitis Suppurativa Physician Global Assessment; *q1w*, every 1 wk; *q2w*, every 2 wk; *q4w*, every 4 wk.

∗ Reported dosages from the most recent follow-up visit.

† Patient discontinued dual therapy following the development of generalized xerosis and eczematous eruption.

‡ Recurrent oral thrush in patients on chronic antibiotics for other medical conditions.

We report herein the largest case series to date to evaluate concomitant biologic therapy in HS. Patients on concomitant biologic therapy demonstrated improved disease control after failing anti-TNF or anti-interleukin 17 monotherapy. Serious adverse events were not observed. Two patients developed suspected psoriasiform dermatitis, a paradoxical skin reaction associated with various biologic monotherapies. These reactions were managed with topical agents and did not require treatment discontinuation. Two patients were older than 65 years, suggesting that combined biologic treatment may be tolerated in older adults.

This study is limited by its retrospective design, sample size, and limited follow-up. Because HS is a chronic, relapsing, and remitting disease, isolated time points may not fully capture overall response; to address this, we incorporated both patient- and physician-reported outcomes. Despite limitations, our findings provide preliminary evidence supporting concomitant anticytokine therapy as a therapeutic alternative for severe, refractory HS. Prior case reports have described improvement with other dual biologic regimens,^4^^,^^5^ and emerging bispecific agents further highlight the growing interest in multipathway therapeutic regimens.

## Conflicts of interest

Dr Naik has received consulting fees from Abbvie, Incyte, Medscape, Sonoma Biotherapeutics, Union Chimique Belge (UCB), Moonlake TX Immunotherapeutics, and Novartis; a grant from Union Chimique Belge (UCB); and holds shares in Radera, Inc. She is a member of the JAMA Dermatology Editorial Board and President of the Hidradenitis Suppurativa Foundation. Drs Bastian, Bizuneh, and Amerson have no conflicts of interest to declare.

## References

[1] Kimball A.B., Okun M.M., Williams D.A. (2016). Two phase 3 trials of adalimumab for hidradenitis suppurativa. N Engl J Med.

[2] Kimball A.B., Jemec G.B.E., Alavi A. (2023). Secukinumab in moderate-to-severe hidradenitis suppurativa (SUNSHINE and SUNRISE): week 16 and week 52 results of two identical, multicentre, randomised, placebo-controlled, double-blind phase 3 trials. Lancet.

[3] Kimball A.B., Jemec G.B.E., Sayed C.J. (2024). Efficacy and safety of bimekizumab in patients with moderate-to-severe hidradenitis suppurativa (BE HEARD I and BE HEARD II): two 48-week, randomised, double-blind, placebo-controlled, multicentre phase 3 trials. Lancet.

[4] Naik H.B., McGinness A., Shinkai K. (2018). Concurrent anticytokine biologics for the management of severe hidradenitis suppurativa: are they safe and effective?. Cutis.

[5] Yu Y., Li J., Liu H., Sun Y., Zhang F. (2025). Dual Blocking of IL17A and IL36R for the treatment of refractory hidradenitis suppurativa: a case report. Biologics.

